# Skills program for awareness, connectedness, and empowerment: A conceptual framework of a skills group for individuals with a psychosis-risk syndrome

**DOI:** 10.3389/fpsyt.2023.1083368

**Published:** 2023-03-02

**Authors:** Tina Gupta, Ligia Antezana, Christian Porter, Tushita Mayanil, Lauren M. Bylsma, Michael Maslar, Leslie E. Horton

**Affiliations:** ^1^Department of Psychiatry, University of Pittsburgh School of Medicine, Pittsburgh, PA, United States; ^2^The Family Institute at Northwestern University, Evanston, IL, United States

**Keywords:** clinical high-risk, psychosis-risk, group therapy, integrative skills group, heterogeneity, dialectical behavioral therapy

## Abstract

Intervention strategies for those diagnosed with psychotic disorders such as schizophrenia can be effective in reducing symptoms and improving quality of life. While strides have been made in developing prevention and intervention strategies earlier on in the disease progression, among those at clinical high-risk (CHR) for psychosis, challenges with heterogeneity can limit symptom and diagnosis specific treatment. Here, we discuss a newly developed therapy skills group called the Skills Program for Awareness, Connectedness, and Empowerment (SPACE) that integrates different types of behavioral skills – standard and radically open dialectical behavioral therapy as well as cognitive behavioral therapy – for CHR youth between the ages of 13–18 years. With the diathesis-stress framework serving as a foundation, the intervention is divided into three stages. These stages target specific signs and symptoms contributing to the progression of CHR symptoms. Stage 1 targets stress (with the goal of developing awareness and reducing distress), stage 2 targets self-disturbances (with a goal of increasing self-connectedness), and stage 3 targets social connectedness (with a goal of improving social domains of functioning). The focus of this article is to introduce the theoretical framework underlying the pilot skills group and discuss ongoing progress.

**Clinical Trial Registration:**

NCT05398120; https://clinicaltrials.gov/ct2/show/NCT05398120.

## 1. Introduction

Interventions for those diagnosed with psychotic disorders are well-known, with standard approaches including psychotropic medication, psychosocial treatments, exercise, and family-based interventions (1–6). While these interventions are critical for this group, there are benefits in taking a more developmental approach, intervening before the emergence of psychosis. Individuals at clinical high-risk (CHR) for developing psychosis show signs and symptoms suggestive of possible conversion to psychosis, with approximately 25% of these cases developing psychosis within a 2–3-year period [(7–9)]. This group tends to endorse positive symptoms (e.g., hearing whispers and seeing shadows), negative symptoms (i.e., reductions in motivations and behaviors), cognitive decline, high rates of comorbid diagnoses such as depression and anxiety, and impairments in social and role functioning (8–11). Importantly, no two individuals are alike in their clinical presentations, which can make applying interventions particularly challenging for this group (11). Yet, intervening early on in the course of psychosis progression can perhaps alter an individual’s psychological trajectory.

While psychosocial interventions for those with CHR syndrome have shown efficacy in reducing symptoms [see (12)], there are still efforts needed to develop and refine interventions given some meta-analytic evidence suggesting an absence of robust effects (13). These findings could be related to several factors including a small number of registered clinical trials, research design and methodological issues, barriers due to mental health stigma, access to clinical care, and/or duration of untreated illness. Clinical heterogeneity, common in this group (11), may also be interfering with treatment progress. Clinical heterogeneity is the notion that individuals can experience unique clinical presentations and perhaps a one size fits all approach to treatment may not be effective (11, 14). For example, in the case of CHR symptoms, one individual may present with unusual thoughts, auditory perceptual aberrations, social anhedonia, and anxiety while another individual may present with visual perceptual aberrations, avolition, and depression.

Here, we discuss the conceptual framework and ongoing progress of a newly developed group intervention called the Skills Program for Awareness, Connectedness, and Empowerment (SPACE) that combines skills from established cognitive behavioral interventions. This integrative skills group may have the potential to address some of the challenges with clinical heterogeneity that can make applying effective treatments for CHR youth particularly difficult. The focus of the group is on common CHR symptoms that may contribute to the emergence of psychosis and/or other psychopathology over time that fall into the categories of (1) difficulty coping with stress (15–17), (2) impairments in self-connectedness (e.g., challenges with self-concept, negative beliefs about self, defeatist beliefs, disrupted identity formation) (18–21), and (3) social impairments (e.g., difficulties developing and maintaining relationships as well as loneliness) (10, 22–24). Furthermore, while this pilot study is a registered clinical trial that has begun recruitment, we hope to introduce this group in this article with the additional goal of incorporating feedback and input from the field in future iterations of the group design.

The three targets of our intervention - stress, self-connectedness, social connectedness - are expanded upon further below. We then introduce our conceptualization for the skills group. Following, group details are provided and then benefits, challenges, and considerations for future work/limitations are discussed.

### 1.1. Stress

Research investigating risk markers of psychosis conceptualize the etiology of psychosis from a diathesis-stress framework. This hallmark model suggests that interactions between genetics such as familial risk and acquired vulnerability such as prenatal insults can interact and form a constitutional vulnerability, termed the diathesis. Stressors (e.g., psychosocial stress, family environment, neighborhood deprivation, negative life events), and atypical neuro-maturational processes can interact with the diathesis, possibly contributing to the emergence of psychosis-risk symptoms and eventual transition over time (25–27). The updated neural diathesis-stress model suggests that those with a likelihood of developing psychosis may be more sensitive to stressors due to HPA axis abnormalities which can impact dopaminergic and glutamatergic pathways in the brain (25). In fact, elevated cortisol secretion, a correlate of stress reactivity, is suggested to contribute to the emergence of positive symptoms (26, 28). Similarly, higher levels of stress have been found to relate to depressive symptoms as well in this group (29). Individuals with CHR syndrome tend to exhibit less effective coping strategies (30–33) and tend to rate higher levels of stress from situations and events compared to those whose psychosis-risk symptoms remitted (33). This pattern is also present in those experiencing a worsening of symptoms longitudinally (34). Given adolescence is a time of change with increasing responsibilities and demands, and the importance of peer relationships and sensitivity to social related stress become more relevant, the ability to find effective ways to cope with stress is imperative. This need is further bolstered by evidence indicating that effective coping strategies are associated with less severe CHR symptoms (31).

### 1.2. Self-connectedness

Self-connectedness is a term that refers to the nature in which individuals feel connected to and understand themselves. There are several factors that can contribute to and enhance self-connectedness. This includes understanding one’s experiences, challenging negative self-talk and defeatist performance beliefs, identifying areas that bring meaning and purpose to one’s life through pinpointing values, and improving self-esteem. The way individuals view and understand themselves and their experiences has been of research and clinical interest for decades (35) and is gaining increasing attention in those at risk for psychotic disorders. For example, there is evidence that individuals with CHR syndrome report more negative beliefs about themselves when compared to their typically developing peers (17). Additionally, there is evidence of more defeatist performance beliefs which refer to negative beliefs about one’s abilities to perform in goal-directed activities (36). Drawing from traditional cognitive behavioral models (37), when reinforced and repeated, these beliefs may contribute to the emergence of negative symptoms (i.e., reductions in emotions, behaviors, and motivation) which is a separate and independent dimension from positive symptoms. However, it is important to note that these beliefs are also related to positive symptoms and transition to psychosis. For example, one study of 765 individuals with CHR syndrome and 280 healthy controls found those that transitioned to psychosis tended to have more maladaptive negative self-schemas at the time of transition (38). Furthermore, there is evidence that endorsement of defeatist beliefs is related to neurocognitive impairment in patients with schizophrenia (37). Perhaps targeting neurocognitive impairment may decrease defeatist beliefs by increasing cognitive flexibility (e.g., the ability to flexibly evaluate and challenge thoughts). Altogether, teaching adolescents and young adults tools and techniques to improve their relationships with themselves may be useful and perhaps even a protective factor in the context of the pathogenesis of psychosis.

### 1.3. Social connectedness

Feeling integrated and having social relationships is critical for overall functioning and quality of life. It has been long established that social related impairments are characteristic of those with CHR syndrome (10, 22, 39, 40). For example, evidence suggests that those with CHR syndrome have fewer social relationships and report experiencing loneliness more often than typically developing peers (23). Furthermore, studies indicate that those with CHR syndrome tend to have lower levels of social support and are overall more isolated (10, 29). Social support predicts clinical course in CHR groups [e.g., (23, 29)]. Social connectedness could reduce stress and protect an individual from poor clinical outcomes such as worsening of positive symptoms and transition to psychosis. Interestingly and of relevance to the current group skills intervention, it is perhaps possible that challenges with emotional awareness and regulation may be one mechanism underlying these difficulties (41). Social functioning is a predictor of conversion to psychosis (42) which highlights the importance of targeting social domains in this group. It is also possible that comorbid diagnoses such as depression and anxiety disorders interfere with social functioning and further reinforce social isolation and withdrawal (43–47). Additionally, with the accumulating evidence pointing towards increased rates of bullying among this group (48–51), there may be opportunities to refine social related interventions that can target a range of social skills. For example, perhaps training in both verbal *and* nonverbal social signaling could be useful, as others have shown with social-cognitive interventions such as Cognitive Enhancement Therapy (52). Social development is critical during adolescence and young adulthood period as these skills may influence several domains of functioning throughout one’s life. Social connectedness and support may also serve as a means to reduce stress and as a result, may protect an individual from later psychopathology.

### 1.4. Psychosocial treatments

Current early intervention strategies for CHR youth include cognitive behavioral therapy (CBT) implemented in both individual and group formats (12). CBT applied to those with CHR syndrome draw heavily from the already developed and established CBT intervention for psychosis (53). CBT models tailored for this population may be especially well-suited to address positive symptoms by providing targeted psychoeducation and directly addressing interpretations of unusual experiences by considering the role of thoughts, feelings, and behavior patterns. To date, there is some evidence for the efficacy of CBT in CHR groups. For example, in one study, Addington et al. (54) implemented CBT in a sample of 51 CHR individuals and observed improvements in positive symptoms, which are central and diagnostic of CHR. However, improvements were not observed in negative symptoms or social functioning. More recently, one group introduced a transdiagnostic cognitive-behavioral treatment for adolescents at high-risk for serious mental illness (schizophrenia and bipolar disorder) which has shown promise (55). Even so, work is needed to improve early intervention and prevention strategies. There may be utility in expanding intervention outcomes beyond transition rates to include additional targets and broadening skills taught in this context.

One novel set of intervention skills that has yet to be tested is the efficacy of dialectical behavioral therapy (DBT) group therapy skills for CHR youth. DBT, originally developed by Dr. Marsha Linehan (56), aims to reduce difficulties with several processes including emotion dysregulation and stress. Standard adult DBT skills have been adapted for adolescence as well (57). A related third-wave DBT intervention, Radically Open (RO) DBT, was recently developed for targeting excessive self-control or overcontrol that can contribute to challenges with social connectedness (58). Overcontrolled tendencies are transdiagnostic in nature and these characteristics include cognitive inflexibility, challenges with emotional expression, increased threat sensitivity, reduced reward processing, and loneliness. Since the development of both standard adult and adolescent DBT, and RO DBT, studies find the application of these interventions can lead to improvements in symptoms and outcomes (e.g., stress, interpersonal difficulties, regulating emotions, etc.) across different psychological disorders and processes (58–60). Both adult and adolescent standard and RO DBT target processes relevant to those with CHR syndrome (e.g., anxiety and depression, social impairment, threat sensitivity, emotional expression).

### 1.5. SPACE group

The SPACE group is a newly developed 21-week skills group that teaches individuals with a CHR syndrome skills to combat challenges with the described domains – stress, self-connectedness, and social connectedness. This group is a clinic-based pilot group expected to recruit a total of 16 individuals with a CHR syndrome (attrition is considered in this final, expected N; please also note N was decided based on current recruitment flow within the clinic). This group is not a randomized control trial (RCT) but is a naturalistic design which may enhance generalizability to other community clinic settings. Please see the “Methods and analysis” section below for more information regarding group details.

## 2. Methods and analysis

### 2.1. Aims and hypotheses

It is important to note that all hypotheses are exploratory given the focus on feasibility and the small sample size. Primary aims are to examine whether implementing a skills group as such is feasible by assessing measures such as dropout rates, weekly attendance, and group satisfaction surveys completed by both clinicians and group members. Secondary outcomes include examining changes in central symptom targets (e.g., stress, self-disturbances, and social connectedness). A third aim is to investigate whether there are changes in psychosis-risk conversion scores over the course of the group. A fourth aim is to investigate auxiliary symptoms that may be more indirectly targeted by the skills group (e.g., self-stigma, positive symptoms, negative symptoms, comorbid depression, and emotion dysregulation). With these aims, it is predicted that a skills group integrating components of different types of DBT (e.g., adolescent, RO) as well as CBT will be feasible. Furthermore, it is predicted that reductions in symptoms and increases in functioning will be observed following the intervention.

### 2.2. Participants

Individuals already receiving services at the Hope Team (website: https://www.hopeteam.pitt.edu) at the University of Pittsburgh Medical Center (Leadership Team: Drs. Leslie Horton, Lauren Bylsma, and Tushita Mayanil) are offered the option to participate in the skills group. Individuals are invited if they are between the ages of 13–18 years. The Hope Team provides individual therapy to those with CHR syndrome drawing on different therapeutic modalities but anchored primarily in CBT. Outcome data for the group are collected pre-post and midway intervention. Individuals can participate in the group if they meet for a CHR syndrome based on criteria outlined in the Structured Interview for Psychosis-Risk Syndromes (SIPS discussed below) (61, 62). This includes meeting criteria for brief intermittent psychotic syndrome (i.e., brief or intermittent frankly psychotic symptoms), attenuated positive symptom syndrome (i.e., recent attenuated positive symptoms), and/or genetic/schizotypal inclusion with deterioration in functioning. Individuals are excluded if they have a history of meeting criteria for a psychotic disorder or develop psychosis over the course of the group. Individuals are able to participate in the study if they have had exposure to CBT or DBT skills given that the skills in this group are adapted for CHR specific symptoms. As mentioned, the reason for this is to adopt a naturalistic design and enhance generalizability to other community settings in this iteration of the group. Furthermore, we collected information on whether individuals were on medications or were receiving additional therapeutic services, but this was not an exclusion criterion of the study. Caregivers are offered the option to participate in separate parent-specific sessions monthly which include discussion of the skills and how parents can help to strengthen and generalize skills. Parents are also asked to complete occasional questionnaires. However, parent participation is not a requirement of the study. Given that this is a pilot study and to facilitate recruitment flow, the group format is rolling admission.

### 2.3. SPACE group stages

**Figure 1:**
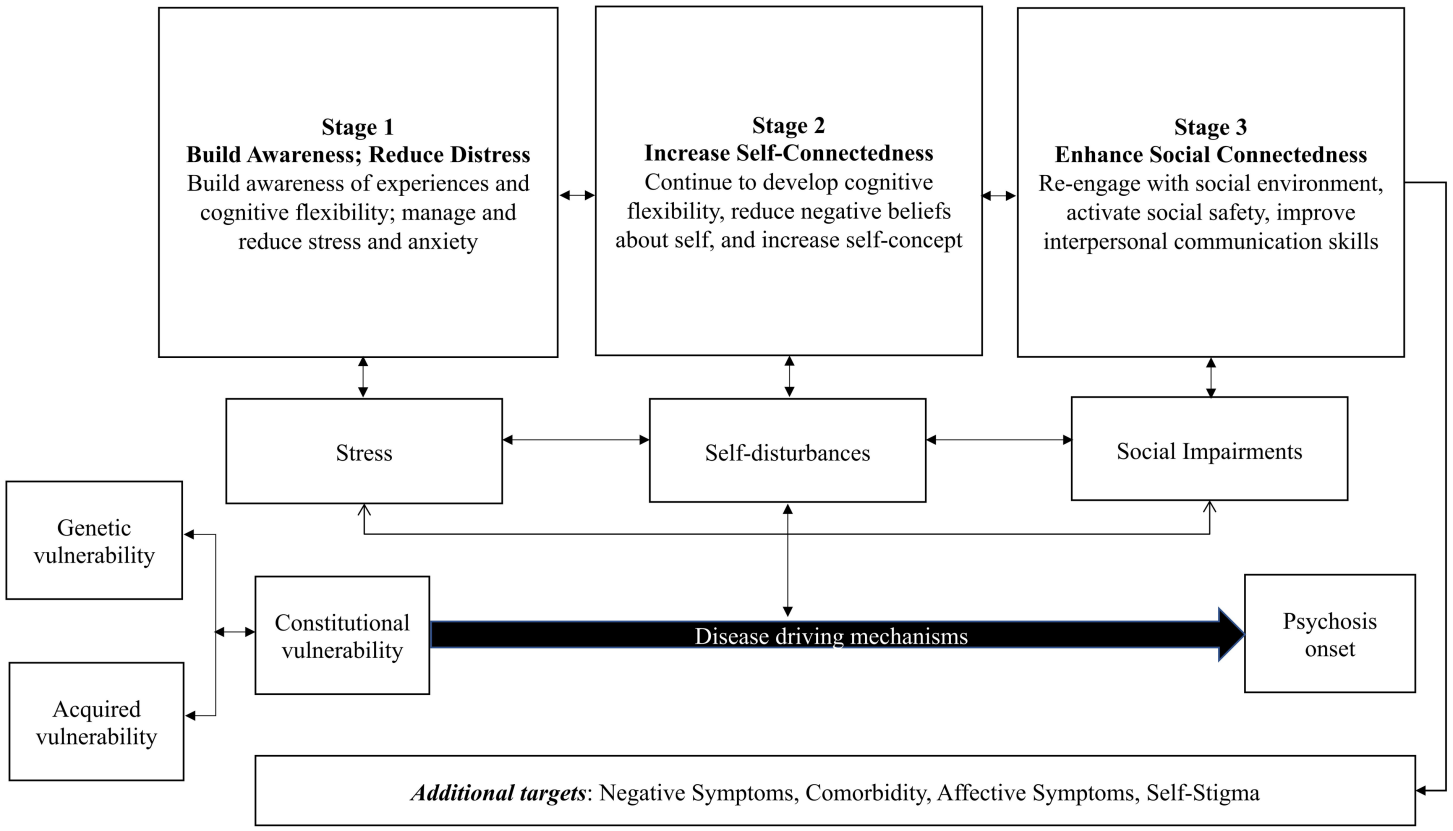
SPACE group conceptual model. The diathesis stress model posits that the onset of psychotic disorders such as schizophrenia can occur through the interaction between genetics and acquired vulnerability. This interaction can lead to a constitutional vulnerability, the diathesis, that can further interact with stress, self-disturbances, and social impairment, possible disease driving mechanisms (amongst others not listed here due to the focus of the framework). The conceptual framework suggests taking an integrative approach to group therapy by targeting stress, self-disturbances, and social impairments could be useful. Furthermore, integrating skills from standard Dialectical Behavioral Therapy [DBT; (68, 69)] such as mindfulness and distress tolerance skills (as in Stage 1 and 2), Cognitive Behavioral Therapy for those at Risk of a First Episode Psychosis [(70); as in Stage 2], and Radically Open DBT skills [(71); as in Stage 3] may address challenges with heterogeneity observed in treatment studies.

The group was developed drawing from the diathesis stress model (27) including modern conceptualizations of this framework (63) and considering disease driving disease driving mechanisms that contribute to worsening of symptoms. Furthermore, this group was developed in an effort to integrate evidence-based practices that currently exist for a range of targeted symptoms to address heterogeneity. The group is structured keeping traditional DBT skills group formats as a foundation, with mindfulness in the beginning of the group therapy session, followed by home practice review, and ending with new skills teaching and new home practice assignment. The group includes three stages that are approximately 7 weeks each and are intended to target specific disease mechanisms (see Figure 1). In the first stage, which draws from adult and adolescent standard DBT skills (e.g., mindfulness, distress tolerance skills), the goal is for an individual to develop an awareness of their own experiences and reduce challenges managing and coping with stress/anxiety. This includes acquiring skills such as mindfulness to help build awareness of experiences and learn to identify when one is feeling stress and intervene with appropriate distress tolerance skills. Importantly, if an individual is able to develop awareness of problematic experiences and reduce stress, then perhaps this individual may have more cognitive flexibility for stage 2 which is focused on increasing one’s self-connectedness through cognitive intervention. Stage 2 involves helping individuals to build awareness and understanding of experiences, focusing on ways to challenge unhelpful thinking patterns and defeatist beliefs, identify values, and improve problem solving skills to live more in line with one’s values. This stage integrates CBT skills (e.g., CHR psychoeducation, cognitive biases, thought records) (64) and components of adult and adolescent standard DBT’s emotion regulation module (e.g., problem solving, accumulating positive emotions). The third stage is built on the notion that once awareness is increased, stress is more effectively managed, and an individual has a stronger sense of their identity as well as more cognitive flexibility, they can “look outward” and focus on building skills to strengthen social connectedness. Stage 3 skills draw from RO DBT. This involves helping individuals to learn how to manage their social safety system when they feel threat activated in a social situation, understanding rejection, improving social signals, and strengthening interpersonal communication. Skills drawn from CBT/DBT are adapted to include discussions of how these skills can be useful for CHR symptoms specifically.

### 2.4. SPACE group details

**Figure 2:**
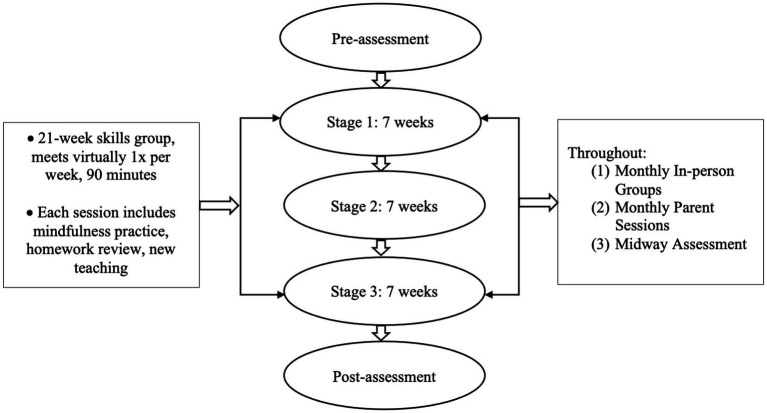
Schematic depicting SPACE group details. Stage 1 goal = Build Awareness; Reduce Distress, Stage 2 goal = Increase Self-Connectedness, Stage 3 goal = Enhance Social Connectedness.

The 21-week clinic-based skills group meets one time per week. This frequency was chosen in order to have a consistent schedule and also balance the need to ease participant burden because adolescents participating are often attending school and extracurricular activities and parents, who are often providing transportation, technological support, and encouragement are managing their own schedules as well (e.g., work). Furthermore, this weekly frequency is in line with some previous work (65, 66) although this is relatively understudied. Additionally, the use of standalone DBT skills is consistent with previous work in the literature (67). The group occurs on a weekday evening for 90 min. The group is a hybrid format which was developed with the feedback of members preferences to be virtual each week with one-monthly in-person session in place of one of the virtual sessions. While the goal was to be responsive to the group members feedback, it is important to acknowledge that there are limitations of the hybrid approach. For example, with the virtual session approach, there may be less group cohesion and engagement (e.g., turning off the camera, walking away from the screen) and increased distractions (e.g., urges to browse the internet, text). However, some benefits of virtual sessions include the ability for individuals to access care regardless of location. Please also note given the changing circumstances of the COVID-19 pandemic, we are working to incorporate more in-person sessions, with virtual as an option if needed for some members. Additionally, the group has 2–3 co-leader therapists. The group is structured so that it begins with a brief mindfulness practice, homework review, and ends with a new teaching. The new skill teaching involves integrating a balance of didactic (orienting as to why the skill is important and how it can help the target discussed), discussion (with an emphasis on how the skill can be useful for the CHR specific intervention target and symptoms), and skill practice. Throughout the skills group teachings, we refer to CHR experiences as “*extraordinary experiences*” in line with CBT interventions for this group (64). Furthermore, parent sessions occur monthly and involve reviewing the skills taught that month, discussing how parents can support skills strengthening and generalization, and problem solving any obstacles. Please see Figure 2 for skills group details.

An important point to discuss is the current group differs from DBT skills groups in many ways. For example, as mentioned, the current group draws from both standard *and* RO DBT in efforts to compile skills that could be useful for the targets discussed and the broader CHR syndrome. These skills are chosen based off of theoretical models (e.g., diathesis stress model) and the CHR literature. As a result and as shown in Table 1, not all DBT skills are implemented, making this group a *DBT informed* skills group. This is in efforts to keep the group focused and specific to CHR related symptoms (e.g., targets that are related to positive symptoms, clinical course, and/or transition to psychosis). Furthermore, the skills group does not utilize the interpersonal skills section from adult and adolescent standard DBT skills but instead draws from RO DBT to address the social connectedness target given the emphasis on threat activation with the RO approach. While DBT targets emotion dysregulation, this skills group is intended to target symptoms suggested to contribute to worsening of positive symptoms and/or transition to psychosis. Additionally, each skill taught in the group is anchored around discussions on how the skill can be useful for reducing CHR specific symptoms in the context of the noted domain (e.g., stage 1: reducing stress). For example, distress tolerance skills are discussed in the context of the diathesis stress model and the notion that stress can worsen CHR symptoms is emphasized in the teaching. A common discussion point is how to manage distress from CHR symptoms which is particularly relevant for stage 1. However, the challenges with managing distress from experiences extends beyond stage 1 and into the other two stages of the group (e.g., how to manage distress that interferes with self-connectedness and social connections).

**Table 1:** Skills taught in each stage of SPACE group.

Skills Taught	Skill Orientation
Stage 1: Build awareness: Reduce distress	
Mindfulness, three states of mind, what skills, how skills, distract with wise mind ACCEPTS, self-soothe, IMPROVE the moment, TIPP	Adult and Adolescent Standard DBT
Stage 2: Increase self-connectedness	
Psychoeducation of extraordinary experiences, cognitive biases and thinking mistakes, challenging extraordinary experience-related thoughts, dialectics/validating self, problem solving, accumulating positive emotions short and long term, build mastery, cope ahead, PLEASE	CBT for Those at Risk of a First Episode Psychosis and adult and adolescent standard DBT
Stage 3: Enhance Social Connectedness	
Activating social safety system, tribe matters: understanding rejection and self-conscious emotions, social signaling matters, ROCKs ON, PROVES, enhancing social connectedness Part 1 and 2	RO DBT

Skills are drawn/adapted from adult and adolescent standard Dialectical Behavioral Therapy (DBT) skills manuals (68, 69), Cognitive Behavioral Therapy for Those at Risk of a First Episode Psychosis (70), and Radically Open (RO) DBT skills manual (71); activities, contributing, comparisons, emotions, pushing away, thoughts, sensations (ACCEPTS); imagery, meaning, prayer, relaxation, one thing in the moment, vacation, encouragement (IMPROVE); temperature, intense exercise, paced breathing, progressive muscle relaxation (TIPP); physical illness, balance eating, avoid mood-altering drugs, balance sleep, get exercise (PLEASE); resist the urge to control others, identify your goals with openness, clarify the effectiveness goal that is priority, practice kindness, consider other’s needs (ROCKs ON); provide a brief description, reveal emotions, acknowledge other person’s needs, use your valued goals, practice self-enquiry (PROVEs).

### 2.5. Primary and secondary outcome measures

As mentioned, all individuals were assessed using the SIPS interview which is the diagnostic interview used to identify CHR syndromes (61, 62). In this interview, questions are asked assessing positive symptom domains (unusual thought content/delusional ideas, suspiciousness/persecutory ideas, grandiose ideas, perceptual abnormalities/hallucinations, disorganized communication). Examples of questions include *Have you felt that you are not in control of your own ideas or thoughts? Do you ever feel your eyes are playing tricks on you?*

Feasibility is assessed by collecting weekly information on attendance and group satisfaction (Modified Quick Lecomte & Leclerc Scale), group engagement and prosocial behaviors (Participation Scale) (72) and co-leader fidelity measures. Additional feasibility measures collected at baseline, midway, and post group include the Satisfaction with Therapy and Therapist Scale (73). Furthermore, group members and parents complete a brief group survey assessing motivation and goals across timepoints.

Secondary outcomes include direct targets of the group. First, different components of stress are assessed using the Perceived Stress Scale (74), Beck Anxiety Scale (75), Child and Adolescent Mindfulness Measure (76), and Avoidance and Fusion Questionnaire for Youth (77). Second, self-connectedness is assessed using the Defeatist Performance Attitudes measure (78), Cognitive Insight Scale (79), and self-stigma measure [Internalized Stigma of Mental Illness measure (80)]. A test of cognition is collected as well - the Hopkins Verbal Learning Test Revised (81) – in order to assess cognitive changes from learning skills in this section (e.g., cognitive restructuring). Third, social connectedness is measured using the Social and Role Functioning scale (82), and (the Social Connectedness Scale) (83).

Other outcomes include negative symptoms (Negative Symptom Inventory – Psychosis-Risk; (84)), depression [Beck Depression Inventory (85)], NAPLS psychosis risk calculator scores (86), quality of life [Lehman Quality of Life Functional Assessment, (87)], positive symptoms [SIPS, Prodromal Questionnaire (88)], emotion regulation [Emotion Regulation Scale, (89)], trauma [Child Trauma Questionnaire (90)], and coping styles [Youth Over and Undercontrol Measure, (58)]. Please note understanding symptoms such as negative symptoms and depressive changes in response to the intervention can inform our understanding of areas of convergence and divergence between the two constructs, which is an ongoing research question in the field (17, 91–93).

### 2.6. Statistical considerations and data analysis plan

Given this is a novel, pilot intervention, the current analyses will be exploratory in nature. Linear mixed-effects models, which will account for attrition, will be used to assess changes within each stage and changes over time in behavioral measures; timepoints are baseline, midway, and post-intervention.

### 2.7. Trial status

Recruitment for baseline assessments began April 26^th^, 2022. The first group session occurred May 10^th^, 2022, and at the time of re-submission, the group is in round 2 of stage 1 teachings. Currently, there are 5 active members in the group. Additionally, there have been a total of 6 caregiver sessions. Generally, the current sample of individuals are white, younger adolescents endorsing moderate to moderate–severe levels of positive symptoms, and moderate to average levels of social and role functioning.

## 3. Discussion

As discussed, this group intervention has the ability to target signs and symptoms (i.e., stress, challenges with self-connectedness, and impairments in social connectedness) contributing to worsening of symptoms or even possible conversion to psychosis. However, there are additional benefits, possible challenges, and considerations for future directions that are useful to discuss which will be the focus of this next section.

### 3.1. Possible benefits

A possible benefit of the skills intervention is the integration of different behavioral skills that are useful for improving many different psychological processes. DBT skills groups are effective for a wide range of psychiatric illnesses including adults and adolescents with suicidal and self-injurious behaviors, those with eating disorders (94), individuals struggling with addictive behaviors among those with alcohol use disorder (95), and individuals with anxiety and depressive disorders (96–99). The breadth of symptoms DBT may address is particularly relevant for those with a CHR syndrome given that this group, as mentioned, is characterized by unique clinical presentations. Additionally, emotion dysregulation and suicidality are characteristic of some individuals that are identified with the CHR syndrome which DBT is effective for improving (100–105). Furthermore, there is evidence that CBT skills are effective for those with CHR syndrome, with a recent systematic review and meta-analysis (106) suggesting reductions in transition rates and positive symptoms. However, there were no beneficial effects for functioning, depression, quality of life, and distress. While not all behavioral skills are beneficial for each individual with CHR syndrome, the wide range of skills taught in this intervention can allow for one to form a “toolbox,” pulling skills that fit an individual’s needs.

Furthermore, these pilot data have the ability to contribute to the growing literature examining the efficacy of RO DBT for adolescents. As mentioned, RO DBT is intended to target processes related to excessive self-control or overcontrol (58). The emphasis on developing skills to enhance social connectedness could be useful for those with CHR syndrome given this group’s social related impairments. A major component of the theory underlying RO DBT is that heightened threat sensitivity can make it more difficult for an individual to enter a social-safety neurobiological system, and engage in prosocial behaviors (58). This heightened sense of stress and threat sensitivity can emerge due to perceptual abnormalities, unusual thoughts, and/or suspicious beliefs, which could contribute to isolation and social withdrawal (25). Thus, there may be skills specifically targeting the stress and the social safety system (e.g., skills to socially signal openness) that could be useful to draw from RO DBT for this group that can enhance social functions. This could be particularly the case given that social signaling is the main mechanism of change in RO DBT (60). However, while RO DBT skills have shown efficacy for treating disorders such as depression and eating disorders, (58, 98) there is still more research underway in which these data have the potential to contribute to particularly given the transdiagnostic nature of these skills. Furthermore, while there is a growing body of evidence applying RO DBT to adolescents (59, 107), there is more work needed.

It is also possible that this skills group may indirectly target insight and strengthen awareness. The ability to change experiences perhaps begins with having awareness of thoughts, emotions, and behaviors. One critical benefit of mindfulness skills is the ability for one to strengthen awareness of experiences. There is evidence that mindfulness interventions are effective in those diagnosed with psychotic disorders such as schizophrenia for a variety of reasons including helping to facilitate relaxation, reduce stress, and decrease symptoms (108–110). While mindfulness skills and interventions are not well understood in terms of efficacy for individuals with CHR syndrome, there are some pilot studies suggesting integrating mindfulness is feasible in this group (108).

This group could also have beneficial impacts on self-stigma. Adolescence can be an isolating time and individuals may be further isolated by experiences that are different from their peers. There are debates as well as to whether early intervention, in general, may perhaps lead an individual to label themselves in unhelpful ways (111). However, there is also work to suggest that risk communication outweigh these risks (112). Even so, the group format, as opposed to individual therapy alone, can be useful in reducing self-stigma. Self-stigma is a barrier to recovery among those with psychotic disorders but is not well understood before onset (113). Of the work that does exist, there is evidence suggesting that those with CHR syndrome may experience shame related to symptoms (113). Discussing and sharing symptoms with others that may have similar experiences could help to reduce self-stigma, increase feelings of validation, and even foster a sense of resiliency. Furthermore, the use of parent sessions is intended to also provide psychoeducation and support around adolescent group participation; parent participation may also be a path towards breaking down barriers related to self-stigma through increased understanding and communication.

The hybrid approach, in which in-person and telehealth groups are alternated or offered simultaneously, may be useful to combat challenges with access to care. This group intervention has the ability to inform this growing area of research. While the COVID-19 pandemic has caused several challenges, the ability to provide interventions virtually has increased access to treatments and interventions (due to transportation, time constraints, work and school schedules etc.) to those who may otherwise be unable to access in-person treatment. It is important to note that the virtual approach to therapy also has challenges such as assuming individuals have access to a laptop, computer, or phone. This is an area of investigation in which further research is warranted.

### 3.2. Current challenges

While there are many benefits to the group, there are challenges we have encountered which we will discuss now and describe some possible solutions that may be useful for other group interventions to consider. First, recruiting individuals with a CHR syndrome can be difficult. Although we are an established clinic which focuses on CHR assessment and intervention and receive regular referrals from the community, there is a greater lack of psychoeducation about the CHR syndrome in the larger social systems including schools, mental health providers, and family, thus, it can be difficult to recruit group members with CHR symptoms. Furthermore, stigma can hinder recruitment as well. One possible solution for this challenge is using rolling admission, which we have implemented, to maximize group recruitment. An additional challenge we have encountered is that there are times in which group members may need higher levels of care and their participation in this study is no longer appropriate. Relatedly, although a participant is identified as having a CHR syndrome, difficulties related to the central targets of the intervention and/or CHR symptoms may not be the primary concern. For example, there may be more imminent diagnoses or concerns that require more comprehensive treatment (e.g., intensive outpatient). As this study is embedded within a larger health care system, we are able to smoothly facilitate this transition. Additionally, some individuals may feel uncomfortable in a group setting due to perhaps social anxiety, trauma, or paranoia related to their CHR status and other intervention may be necessary prior to or instead of participation.

Regarding challenges with running the group itself, group engagement can be difficult to establish with a new group. To address this, we have found weekly reminders, calendar print outs, and group reminder phone calls are some tools that can be useful. Furthermore, parent sessions are important in that we are able to provide psychoeducation to parents who can further support their adolescent’s group participation. Additionally, the maturity level of the group has fluctuated based on ages and cognitive abilities of those enrolled in group. Adapting and approaching the skills to meet the maturity and cognitive levels of participants is one strategy to combat this issue (e.g., using more basic language, asking members to paraphrase, using homework review to assess understanding of skills and provide feedback). There have also been hurdles related to using a hybrid format. First, there are sometimes technology issues (i.e., internet being slow for group members or leaders) which interfere with the flow of a session. Secondly, virtual formats can interfere with cohesion, as discussed, while in-person sessions, anecdotally, tend to have more active engagement, Interestingly, despite high rates of social anxiety in those with a CHR syndrome (44, 45), we have found that group members are more willing to participate in discussions and exercises as group cohesion and engagement is established over time and this is even more the case during in-person sessions.

### 3.3. Additional considerations for future work and limitations

While this study shows several strengths such as integrating different DBT skills (adult and adolescent standard and RO DBT), the use of a group format, and the targeting of central and auxiliary CHR symptoms in efforts to combat challenges with heterogeneity, there are important limitations to discuss for future work to consider. This includes the use of rolling admission. For the pilot nature of the study, there is rolling admission which means individuals can attend at any point in the group. While there are benefits to this approach such as allowing individuals to receive services immediately rather than having to wait until a certain module, there are drawbacks as well. For example, it may be the case that an individual joins during the self-connectedness module and does not get stage 1 first (e.g., skills for building awareness and reducing distress), thus reducing potential benefits to the ordering of stages suggested by our conceptual model. Furthermore, the hybrid approach may have limitations such as impacts on group cohesion. Additionally, this intervention does not have a comparison group; additional iterations of the group would benefit from a treatment as usual condition. Given that individuals may be receiving other services (e.g., therapy, medication management), and this is not standardized in this study due to pilot/feasibility nature of the approach, it is possible that any improvements may be due to other components of intervention instead of or in addition to group. Furthermore, while skills are being taught pulling from three different modalities, not all skills in each modality are being used which may impact any interpretations regarding the efficacy of DBT skills alone for this group. We also assess verbal memory as a means to understand changes in cognition from skills taught in the self-connectedness section. However, future work should consider applying a more comprehensive battery of cognitive tests.

The current study also includes volunteers from within an existing program which does mimic real-world practice but also limits generalizability (e.g., including individuals who are motivated to participate). Future research and iterations of the group would benefit from including individuals across different programs and perhaps also individuals not already embedded within a CHR clinic. It is also perhaps a limitation of the study that we included individuals on medications and those receiving other therapeutic services. While this is also a strength in that this increases the generalizability of findings, future groups would benefit from assessing medication use and how the intervention is impacted by individuals participating in other therapies. Importantly, our pilot intervention does include a wider age range of 13–18 which may also be a limitation to the study. This age range is in line with the age ranges in CHR studies (114, 115). Furthermore, while the intervention is intended to target clinical heterogeneity and we see this as a strength of this approach, targeting heterogeneity (e.g., taking a broader approach to intervention, teaching skills for several psychological processes) in the sample may also be a limitation that could impact recruitment and results. It is also noteworthy that we did not exclude individuals that have had previous exposure to CBT or DBT given that these skills are adapted to target CHR specific symptoms. However, future iterations of the group will consider the inclusion/exclusion of group members based on previous exposure to CBT and/or DBT modalities. Furthermore, while the intervention is intended to increase our understanding of many symptoms often endorsed by those with a CHR syndrome, it will be particularly important for future iterations of the intervention to examine the relationships between negative symptoms and depression in the context of intervention outcomes. Additional iterations would also benefit from including longer follow up durations of the intervention to examine the impacts of the intervention long-term, transition rates, and retention of skills. One additional important point to consider is that while the intervention focuses on distress, mood, and social functioning, our outcome measures include a battery of measures that assess for CHR symptom changes. While we do have measures assessing for general psychiatric morbidity, future iterations should consider adding more measures that assess psychological processes that are not necessarily unique and specific to CHR symptoms.

## 4. Conclusion

Few psychosocial interventions exist for those with a CHR syndrome. Given that CHR group are heterogenous in nature, one single intervention may not be effective for reducing central and auxiliary symptoms. Deriving from the diathesis-stress framework, we developed a group intervention, SPACE group, for adolescents and young adults ages 13–18 years, which focuses on building skills to reduce stress, increase self-connectedness, and enhance social connectedness. This group includes psychoeducation and integrates evidence-based skills from CBT and DBT to target specific signs and symptoms which are theorized to contribute to the progression of psychosis.

## Data availability statement

The original contributions presented in the study are included in the article/supplementary material, further inquiries can be directed to the corresponding author.

## Ethics statement

The studies involving human participants were reviewed and approved by University of Pittsburgh Institutional Review Board. Written informed consent to participate in this study was provided by the participants’ legal guardian/next of kin.

## Author contributions

TG, TM, LB, and LH developed the study concept and design in consultation with MM. TG drafted the article and all authors contributed to writing and revisions. All authors contributed to the article and approved the submitted version.

## Funding

This work is supported by the Substance Abuse and Mental Health Services Administration (SAMHSA) CHR-P grant number H79SM081196 to the Pennsylvania Office of Mental Health and Substance Use Services (OMHSAS) and T32MH018269 to TG and T32MH018951 to LA.

## Conflict of interest

The authors declare that the research was conducted in the absence of any commercial or financial relationships that could be construed as a potential conflict of interest.

## Publisher’s note

All claims expressed in this article are solely those of the authors and do not necessarily represent those of their affiliated organizations, or those of the publisher, the editors and the reviewers. Any product that may be evaluated in this article, or claim that may be made by its manufacturer, is not guaranteed or endorsed by the publisher.
